# Comparing the long-term clinical and economic impact of ofatumumab versus dimethyl fumarate and glatiramer acetate in patients with relapsing multiple sclerosis: A cost-consequence analysis from a societal perspective in Germany

**DOI:** 10.1177/20552173221085741

**Published:** 2022-03-29

**Authors:** Dominik Koeditz, Juergen Frensch, Martin Bierbaum, Nils-Henning Ness, Benjamin Ettle, Umakanth Vudumula, Kapil Gudala, Nicholas Adlard, Santosh Tiwari, Tjalf Ziemssen

**Affiliations:** 60852 Novartis Pharma GmbH, Nuremberg, Germany; 31322 Hexal AG, Holzkirchen, Germany; Novartis Pharma GmbH, Nuremberg, Germany; 36373 Novartis Ireland Limited, Dublin, Ireland; 135093 Novartis Healthcare Pvt. Ltd, Hyderabad, India; 111826 Novartis Pharma AG, Basel, Switzerland; Novartis Ireland Limited, Dublin, Ireland; Zentrum für klinische Neurowissenschaften, Universitaetsklinikum Carl Gustav Carus Dresden, Dresden, Germany

**Keywords:** multiple sclerosis, disease-modifying therapies, disability progression, societal costs

## Abstract

**Background:**

Evidence suggests that early highly efficacious therapy in relapsing multiple sclerosis is superior to escalation strategies.

**Objective:**

A cost-consequence analysis simulated different treatment scenarios with ofatumumab (OMB), dimethyl fumarate (DMF) and glatiramer acetate (GA): immediate OMB initiation as first treatment, early switch to OMB after 1 year on DMF/GA, late switch after 5 years or no switch.

**Methods:**

An EDSS-based Markov model with a 10-year time horizon was applied. Cycle transitions included EDSS progression, improvement or stabilization, treatment discontinuation, relapse or death. Input data were extracted from OMB trials, a network meta-analysis, published literature, and publicly available sources.

**Results:**

The late switch compared to the immediate OMB scenario resulted in a lower proportion of patients with EDSS 0–3 (Δ − 7.5% DMF; Δ − 10.3% GA), more relapses (Δ + 0.72 DMF; Δ + 1.23 GA) and lower employment rates (Δ − 4.0% DMF; Δ − 5.6% GA). The same applies to late versus early switches. No switch scenarios resulted in worse outcomes. Higher drug acquisition costs in the immediate OMB and early switch scenarios were almost compensated by lower costs for patient care and productivity loss.

**Conclusion:**

Immediate OMB treatment and an early switch improves clinical and productivity outcomes while remaining almost cost neutral compared to late or no switches.

## Introduction

Multiple sclerosis (MS) is a chronic, inflammatory, autoimmune disease of the central nervous system manifesting typically between 20 and 40 years of age and leading to accumulation of disability.^1^ The disease represents an enormous health and societal burden.^2,3^

Current strategies in relapsing MS (RMS), i.e., clinically isolated syndrome, relapsing-remitting MS (RRMS) and active secondary progressive MS (SPMS), initially suggest mildly to moderately effective therapies (e.g. beta interferons, glatiramer acetate [GA], dimethyl fumarate [DMF], and teriflunomide) followed by a switch to highly effective therapies (e.g. fingolimod, natalizumab, ocrelizumab, alemtuzumab, cladribine) in case of insufficient response.^4–6^ The use of highly effective, disease-modifying therapies (DMT) in the early phase of MS is evolving,^7–10^ as an early window of opportunity is assumed, when treatment is most effective and critical to maintain neurological function^11^ and reduce disability progression.^8–10^ In line with this, the early intervention with fingolimod, natalizumab and alemtuzumab in patients with RRMS was associated with a reduced risk of transition to SPMS.^7^

The anti-CD20 monoclonal antibodies ocrelizumab and ofatumumab (OMB) are highly effective DMTs, which selectively deplete B-cells.^12,13^ Ocrelizumab is a humanized antibody and is applied through intravenous infusion,^13^ while OMB is fully human and can be applied subcutaneously.^12^ Treatment with ocrelizumab or OMB significantly reduced the relapse rates compared to standard treatment in the pivotal RMS studies.^14,15^ The early application of OMB revealed a positive benefit-risk ratio in the pivotal ASCLEPIOS trials, which was even more pronounced in treatment-naïve RMS patients.^14,16^ OMB was approved for RMS in August 2020 by the *United States* Food and Drug Administration^17^ and in March 2021 by the European Medicines Agency.^18^

Beyond clinical outcomes, cost consequences are becoming a focal point of drug characterizations,^19^ but have not yet been evaluated for OMB. The objective of the present analysis was to simulate the long-term clinical and health economic effects of an immediate initiation of OMB (at time of first treatment) compared to an early (after one year), a late (after 5 years) and no switch to OMB after standard DMT in RMS patients. For this purpose, a cost-consequence analysis (CCA) was applied. A CCA approach involves wide-ranging assessments of direct and indirect costs as well as various outcomes (consequences). In contrast to cost-effectiveness analyses or cost-utility analyses, outcomes are listed separately, and no cost-outcome ratio is calculated. The CCA provides decision-makers with a comprehensive overview of the impact of interventions, enabling them to form their opinions about the relative importance of costs and outcomes in their particular context.^20^

## Materials and methods

This CCA is a mathematical simulation which combines clinical and health economic data of RMS patients from various sources in a discrete-time Markov model. The model assumes that the patient is always in one of a finite number of discrete health states. Disease improvement or worsening is represented as the risk of transition to another Markov (health) state within a certain time frame (cycle). Clinical and health economic data are assigned to these health states and accumulated over time per patient. The Markov states are based on the Expanded Disability Status Score (EDSS) using integer EDSS values (intermediates were rounded down). The cohort was exposed to the following risks in each cycle: EDSS progression, EDSS improvement, stable EDSS, DMT discontinuation at an EDSS > 6.0 (in line with OMB trials), relapse or death.

Details of the model structure and the input variables are described in the supplement. In brief, the model included patients with RMS and a baseline EDSS of 0–6. The treatment-naïve subpopulation of the combined ASCLEPIOS I and II trials were used for baseline data input. This subpopulation was typical of early RMS with 98.7% RRMS and 1.3% SPMS patients, a mean (± standard deviation, SD) EDSS of 2.3 ± 1.2, a mean (±SD) age of 36.3 ± 9.23 years and 33.0% male patients (Supplementary Table S1). The transition probabilities between EDSS states of the untreated model were based on published British Columbia Natural History data. Annualized relapse rates (ARR) in the natural history model ranged from approximately 0.7 (EDSS ≤ 4) to 0.5 (EDSS > 4) (Supplementary Table S2). The transition probabilities and relapse rates for the treatment-adjusted model were derived from 6-month confirmed disability progression (6-CDP) and relapse rates of a network meta-analysis (NMA), respectively. Details on the resulting NMA including study comparability have been published by Samjoo et al.^21^ The CDP and ARR results included in the model are presented in Supplementary Table S2. Mortality rates were based on the general population mortality,^22^ stratified for gender and age and adjusted using MS population mortality hazard ratios.^23^ Productivity loss data, disability weights of health states, drug- and disease-related costs were identified from published literature and publicly available data sources (Supplementary Tables S3–S6). Costs and effects were discounted at 3% per year. The assignment to an EDSS health state based on the untreated and treated transition probabilities and relapse rates determined clinical and economic outcomes.

### Scenarios

Four scenarios with a time horizon of 10 years were simulated. The two base scenarios evaluated OMB versus standard DMT (DMF or GA) without any treatment switches. Two switch scenarios were defined similar to an analysis of the MSBase registry and the Swedish MS registry recently reported by He et al.^9^ He et al.^9^ analyzed patients who started 0–2 years (early) or 4–6 years (late) after clinical disease onset. The mean time to first high-efficacy therapy was 1.08 years (SD 0.52) in the early and 4.99 years (SD 0.60) in the late treatment group. To allow for comparability with real-life data reported by He et al.^9^ the present simulation accordingly included a scenario with early switch to OMB after one year of treatment with DMF (early DMF/OMB group) or GA (early GA/OMB group) as well as a late switch scenario after 5 years of DMF or GA treatment (late DMF/OMB group and late GA/OMB group).

### Model outcomes

Clinical outcomes included EDSS distribution, time spent in different health states, mean EDSS score over time, progression to immobility (EDSS ≥ 7), and number of relapses. Further clinical outcomes were disability-adjusted life years (DALY), including year's life-lost (YLL) and years lived with disability (YLD). YLL corresponds to the number of deaths multiplied by the remaining age- and sex-specific life expectancy of the general population at the time of death. To calculate the YLD, the number of patients in a particular EDSS state was multiplied by MS-specific weighting factors.

Economic analyses were conducted from the societal perspective accounting for direct and indirect costs regardless of who bears them. Direct costs comprised healthcare costs (DMT acquisition, inpatient care, day care admissions, consultations, tests and investigations, other medications than DMT) as well as costs for services and informal care (community and social services, investments, equipment and aids, informal care). The calculations for informal care costs included resource utilization in time in days. Indirect costs comprised expenses associated with MS-related productivity loss (short-term absenteeism, long-term absenteeism, invalidity, and early retirement). The calculations were based on the productivity outputs (proportion of patients being employed/self-employed, working full-time or receiving invalidity pension). Input data on direct and indirect costs include relapse-related expenses (e.g. healthcare costs, medications, absenteeism). As it remained unclear, to which extent relapse-related costs have already been covered, relapse costs were estimated separately. For this purpose, the quarterly costs reported by Ness and colleagues^24^ were upscaled to annual relapse costs and estimated at 2662 €, taking into account the consumer price indices (Supplementary Table S6).

### Sensitivity analyzes

Univariate sensitivity analyses were performed to determine the impact of drug acquisition costs of OMB at Year 1, 2 and from Year 2 onwards, 6-CDP hazard ratio; cohort size, age, gender; discount rates and annual relapse costs on the model outputs. The factors varied the parameter value by +10% or −10% of the base case value.

## Results

### Clinical outcomes, informal care utilization and productivity output

**Figure 1.**
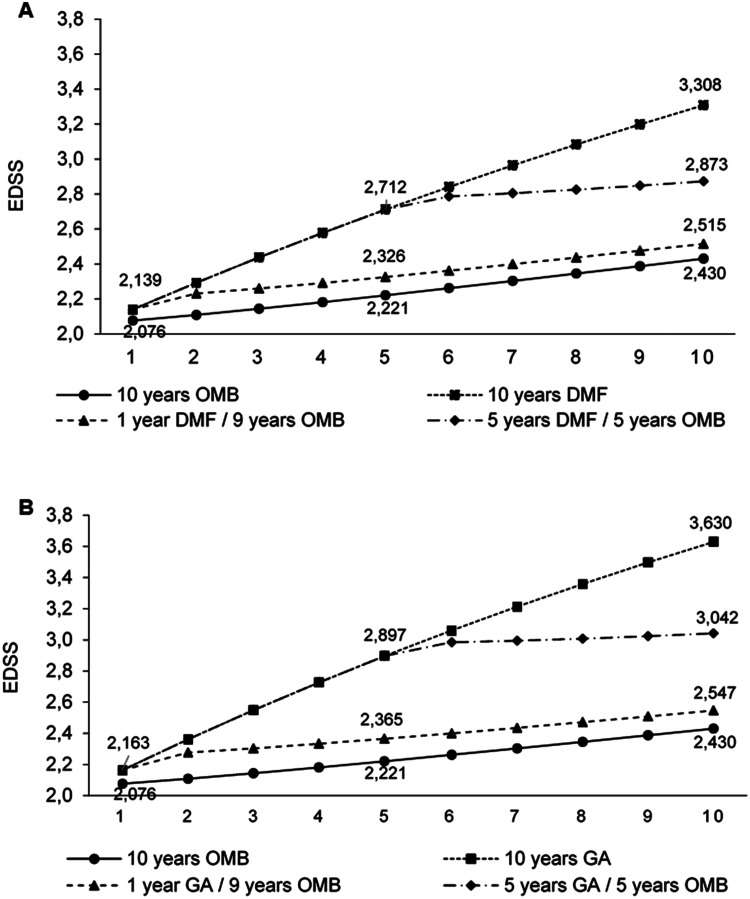
Development of the mean EDSS score over 10 years per scenario; OMB vs. DMF (A); OMB vs. GA (B); DMF, Dimethyl fumarate; EDSS, Expanded Disability Status Scale; GA, Glatiramer acetate; OMB, Ofatumumab.

**Figure 2.**
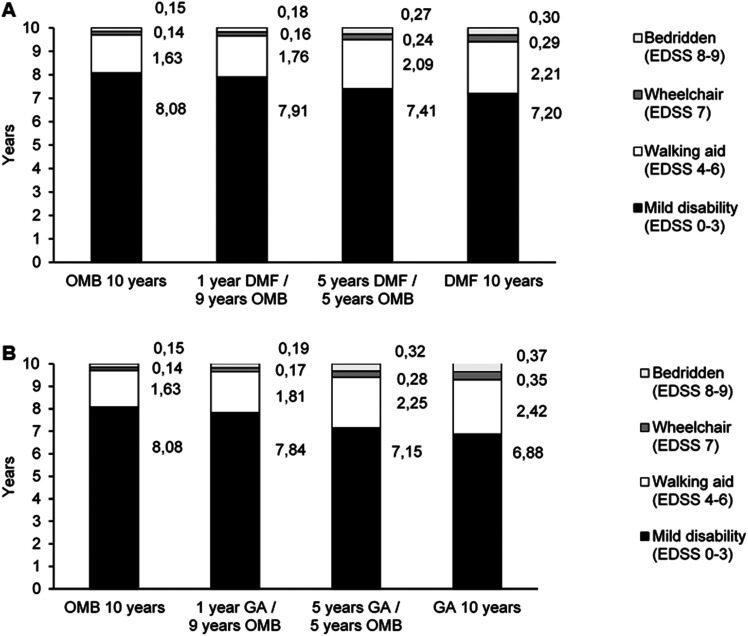
Patient time spent in health states (in years); OMB vs. DMF (A); OMB vs. GA (B); DMF, Dimethyl fumarate; EDSS, Expanded Disability Status Scale; GA, Glatiramer acetate; OMB, Ofatumumab.

Patients immediately treated with OMB had a lower degree of disability after 10 years compared to patients who initially received a standard DMT. In detail, the proportion of patients with no or mild impairment (EDSS 0–3) was lower in the late switch scenario compared to the immediate OMB scenario (DMF/OMB Δ − 7.5%; GA/OMB Δ − 10.3%) while early switch scenarios showed minor differences (DMF/OMB Δ − 1.4%; GA/OMB Δ − 2.0%). Vice versa, the proportion of immobile patients (EDSS 7–9) in the immediate OMB cohort and the early switch scenario were comparable (DMF/OMB Δ + 0.7%; GA/OMB Δ + 0.9%) but increased in the late switch scenario (DMF/OMB Δ + 3.5%; GA/OMB Δ + 4.9%) (Table 1). Mean EDSS after 10 years was lower in the immediate OMB group (2.4) and the early switch groups (2.5 early DMF/OMB and 2.5 early GA/OMB) compared to the late switch scenario (2.9 late DMF/OMB and 3.0 late GA/OMB) (Figure 1(a) and (b)). EDSS distribution over time is presented in Supplementary Figure 2S and 3S. Patients with immediate or early OMB treatment remained in EDSS stages 0–3 for 8 years while those in the late switch scenario remained there for 7 years (Figure 2(a) and (b)).

**Table 1.** Outcomes (OMB vs. DMF/GA).

	Base-Scenario (10/0)^a^	Scenario A (1/9)^b^		Scenario B (5/5)^c^		Base-Scenario (0/10)^a^	
Outcomes	OMB	DMF&OMB Δ^*^	GA&OMB Δ^*^	DMF&OMB Δ^*^	GA&OMB Δ^*^	DMF Δ^*^	GA Δ^*^
***Clinical* * outcomes***							
% Patient distribution in EDSS states							
Mild disability (EDSS 0–3)	76.1%	−1.4%	−2.0%	−7.5%	−10.3%	−14.7%	−20.2%
Walking aid (EDSS 4–6.5)	18.0%	+0.8%	1.1%	+4.0%	+5.4%	+8.2%	+10.7%
Wheelchair (EDSS 7)	2.4%	+0.3%	+0.3%	+1.2%	+1.7%	+2.8%	+4.1%
Bedridden (EDSS 8–9)	3.5%	+0.4%	+0.6%	+2.3%	+3.2%	+3.7%	+5.4%
Immobile patients (EDSS 7–9)	5.9%	+0.7%	+0.9%	+3.5%	+4.9%	+6.5%	+9.5%
DALYs	1.49	+0.07	+0.10	+0.28	+0.40	+0.38	+0.53
YLD	0.86	+0.07	+0.10	+0.28	+0.40	+0.38	+0.53
YLL	0.63	0	0	0	0	0	0
Number of relapse events	2.15	+0.15	+0.26	+0.72	+1.23	+1.33	+2.26
***Informal care utilization***							
Informal care (time in days)	168.38	+12.44	+17.13	+52.42	+74.27	+71.12	+101.50
***Productivity output***							
Employed or self-employed at working age (%)^d^	62.3%	−0.7%	−1.0%	−4.0%	−5.6%	−8.0%	−11.0%
Working full time (%)^e^	36.7%	−0.1%	−0.1%	−0.4%	−0.6%	−0.8%	−1.1%
Receiving invalidity pension (%)^d^	26.5%	+0.6%	+0.8%	+2.8%	+3.9%	+5.4%	+7.6%

DALYs: Disability adjusted life years; DMF: Dimethyl fumarate; EDSS: Expanded Disability Status Scale; GA: Glatiramer acetate; OMB: Ofatumumab; YLD: Years lost due to disability; YLL: Years of life lost; Δ: Difference.

^*^ The delta indicates the difference in outcomes between the comparator arms of the respective scenarios (baseline scenario, scenario A & B) and the intervention group (10 years of OMB administration).

^a^ Base-Scenario: H2H: 10 years of therapy with OMB vs. 10 years of therapy with DMF/GA.

^b^ Scenario A: 1-year therapy with DMF/GA followed by 9 years of treatment with OMB.

^c^ Scenario B: 5 years therapy with DMF/GA followed by 5 years of treatment with OMB.

^d^ Measured in terms of the number of people of working age (retirement age: 67).

^e^ Measured in terms of the number of employees.

The number of DALYs increased with the duration of standard treatment compared to immediate OMB treatment (early switch: DMF/OMB Δ + 0.07%; GA/OMB Δ + 0.10%; late switch: DMF/OMB Δ + 0.28%; GA/OMB Δ + 0.40%) (Table 1). The increase of DALYs depended solely on YLD (Table 1). The analysis also revealed more relapses in the late switch compared to the immediate OMB scenario (DMF/OMB Δ + 0.72; GA/OMB Δ + 1.23), while the early OMB scenario showed minor differences (DMF/OMB Δ + 0.15; GA/OMB Δ + 0.26) (Table 1).

After 10 years, the proportion of patients still employed or self-employed at working age was lower in the late switch scenarios (DMF/OMB Δ − 4.0%; GA/OMB Δ − 5.6%) compared to immediate OMB treatment. More patients received invalidity pension in the late switch compared to immediate OMB scenario (DMF/OMB Δ + 2.8%; GA/OMB Δ + 3.9%). Informal care was utilized to a considerably higher extent in the late switch scenario compared to the group with immediate OMB treatment (DMF/OMB Δ + 52.42 days; GA/OMB Δ + 74.27 days) (Table 1). Differences between early switch and immediate OMB scenarios regarding productivity and informal care use were marginal (Table 1).

The worst results with respect to clinical outcomes, informal care utilization and productivity output were estimated for patients receiving standard DMT throughout 10 years (Table 1; Figures 1(a), (b), 2(a) and (b)).

### Economic outcomes from a societal perspective

For 10 years treatment with OMB, cumulative DMT costs of 145,918 € per patient were estimated. The costs slightly decreased in the early switch scenario (DMF/OMB Δ − 6.3%; GA/OMB Δ − 4.9%) and to a greater extent in the late switch scenario (DMF/OMB Δ − 22.2%; GA/OMB Δ − 15.4%). These decreases were contrasted by higher costs especially in the late and no switch scenario for inpatient care, informal care, community, and social services as well as long-term absence, invalidity and early retirement (Table 2).

**Table 2.** Breakdown of costs (discounted)—costs per patient.

	Base-Scenario (10/0)^a^	Scenario A (1/9)^b^		Scenario B (5/5)^c^		Base-Scenario (0/10)^a^	
	OMB	DMF&OMB Δ%^*^	GA&OMB Δ%^*^	DMF&OMB Δ%^*^	GA&OMB Δ%^*^	DMF Δ%^*^	GA Δ%^*^
Direct costs**							
Healthcare costs (€)							
DMT costs	145.918	−6.3%	−4.9%	−22.2%	−15.4%	−36.9%	−24.3%
Inpatient care	14.963	+4.6%	+6.4%	+17.9%	+24.8%	+23.3%	+32.3%
Day care admissions	1.281	+1.8%	+2.4%	+6.8%	+9.2%	+8.9%	+11.9%
Consultations	10.125	+2.0%	+2.8%	+7.7%	+10.6%	+10.1%	+13.8%
Tests & Investigations	2.866	−0.1%	−0.1%	−0.3%	−0.6%	−0.4%	−0.7%
Medications	5.270	+5.0%	+6.9%	+18.7%	+25.8%	+24.3%	+33.5%
Services and informal care costs (€)							
Community & social services	4.815	+11.2%	+15.4%	+45.9%	+65.3%	+60.1%	+85.9%
Investments, equipment & aids	4.463	+7.6%	+10.4%	+29.2%	+40.8%	+38.2%	+53.5%
Informal care	15.220	+8.4%	+11.5%	+33.0%	+46.4%	+43.4%	+61.1%
**Sum direct costs (€)**	204.921	−2.9%	−1.2%	−9.4%	−2.1%	−17.9%	−5.6%
***Indirect costs***							
Short-term absence	5.617	−0.6%	−0.8%	−1.8%	−2.8%	−2.1%	−3.5%
Long-term absence, invalidity & early retirement	83.932	+3.3%	+4.5%	+12.3%	+17.0%	+16.1%	+21.9%
**Sum indirect cost (€)**	89.549	+3.0%	+4.2%	+11.5%	+15.7%	+14.9%	+20.3%
***Total costs***							
Sum direct/indirect costs (€)^#^	294.470	−1.1%	+0.4%	−3.1%	+3.3%	−8.0%	+2.3%
Relapse costs (€)^#^	5.028	+7.8%	+13.4%	+35.5%	+61.0%	+61.7%	+105.1%
**Total costs (€)^#^**	299.498	−0.9%	+0.6%	−2.4%	+4.3%	−6.8%	+4.0%

DMF: Dimethyl fumarate; DMT: Disease-modifying therapies; GA: Glatiramer acetate; OMB: Ofatumumab.

^*^ The delta indicates the difference in costs between the comparator arms of the respective scenarios (base-scenario, scenario A & B) and the intervention group (10 years of OMB treatment). A negative delta (−) is equivalent to lower costs in the cohorts with permanent DMF administration or delayed therapy initiation compared to the population with permanent OMB treatment. A positive delta (+) equates to lower costs in the intervention group compared to the respective comparator arms.

** Direct costs also include “out-of-pocket” expenses, making it impossible to look at them from a payor perspective.

#: Direct and indirect costs extracted from the referenced data sources include relapse-related expenses, however, it remained unclear, to which extent relapse-related costs were covered. In case of incomplete coverage of relapse costs in the input data, the sum of direct/indirect costs might underestimate MS-related costs. Therefore, a separate estimation of relapse-related costs was included in the total costs. As the sum of direct and indirect costs already include relapse costs at least partially, the total cost estimation represents an overestimation.

^a^ Base-Scenario: H2H: 10 years of therapy with OMB vs. 10 years of therapy with DMF/GA.

^b^ Scenario A: 1-year therapy with DMF/GA followed by 9 years of treatment with OMB.

^c^ Scenario B: 5 years therapy with DMF/GA followed by 5 years of treatment with OMB.

The different scenarios resulted in similar expenditures when direct and indirect costs were summarized (Table 2). Direct and indirect costs amounted to 294,470 € per patient for 10 years of OMB treatment with small differences of Δ − 8.0% to Δ + 3.3% compared to the other scenarios. Taking into account the costs of relapses (5028 €), the total costs for 10 years of OMB treatment amounted to 299.498 € with small differences of Δ − 6.8% to Δ + 4.0% compared to the switch scenarios (Table 2).

The breakdown of cost types per scenario revealed that DMT costs accounted for more than half of direct costs, followed by costs for informal care, inpatient care, and consultations. Indirect costs were dominated by the expenses due to long-term absence from work, invalidity, and early retirement. Approximately two thirds of the total costs were attributed to direct costs and one third to indirect costs. A general cost shifting trend from direct to indirect costs was observed starting from immediate OMB treatment over early to late switch scenarios (Table 3).

**Table 3.** Percentage breakdown of cost types per scenario (OMB vs. DMF/GA).

	Base-Scenario (10/0)^a^	Scenario A (1/9)^b^		Scenario B (5/5)^c^		Base-Scenario (0/10)^a^	
	OMB	DMF&OMB	GA&OMB	DMF&OMB	GA&OMB	DMF	GA
***Direct costs***							
Healthcare costs							
DMT costs^d^	71.2%	68.7%	68.6%	61.2%	61.5%	54.7%	57.1%
Inpatient care^d^	7.3%	7.9%	7.9%	9.5%	9.3%	11.0%	10.2%
Day case admissions^d^	0.6%	0.7%	0.6%	0.7%	0.7%	0.8%	0.7%
Consultations^d^	4.9%	5.2%	5.1%	5.9%	5.6%	6.6%	6.0%
Tests & Investigations^d^	1.4%	1.4%	1.4%	1.5%	1.4%	1.7%	1.5%
Medications^d^	2.6%	2.8%	2.8%	3.4%	3.3%	3.9%	3.6%
Services and informal care costs							
Community & social services^d^	2.3%	2.7%	2.7%	3.8%	4.0%	4.6%	4.6%
Investments, equipment & aids^d^	2.2%	2.4%	2.4%	3.1%	3.1%	3.7%	3.5%
Informal care^d^	7.4%	8.3%	8.4%	10.9%	11.1%	13.0%	12.7%
Sum direct costs^e^	69.6%	68.3%	68.4%	65.0%	65.9%	62.0%	64.2%
***Indirect costs***							
Short-term absence^f^	6.3%	6.1%	6.0%	5.5%	5.3%	5.3%	5.0%
Long-term absence, invalidity & early retirement^f^	93.7%	93.9%	94.0%	94.5%	94.7%	94.7%	95.0%
Sum indirect costs^e^	30.4%	31.7%	31.6%	35.0%	34.1%	38.0%	35.8%
Sum direct/indirect costs	100%	100%	100%	100%	100%	100%	100%
Relapse costs^e^	1.7%	1.9%	1.9%	2.4%	2.7%	3.0%	3.4%

DMF: Dimethyl fumarate; DMT: Disease-modifying therapies; GA: Glatiramer acetate; OMB: Ofatumumab.

^a^ Base-Scenario: H2H: 10 years of therapy with OMB vs. 10 years of therapy with DMF/GA.

^b^ Scenario A: 1-year therapy with DMF/GA followed by 9 years of treatment with OMB.

^c^ Scenario B: 5 years therapy with DMF/GA followed by 5 years of treatment with OMB.

^d^ In proportion to direct costs.

^e^ In proportion to sum direct/indirect costs (Basis: lower cost limit).

^f^ In proportion to indirect costs.

### Sensitivity analyses

**Figure 3.**
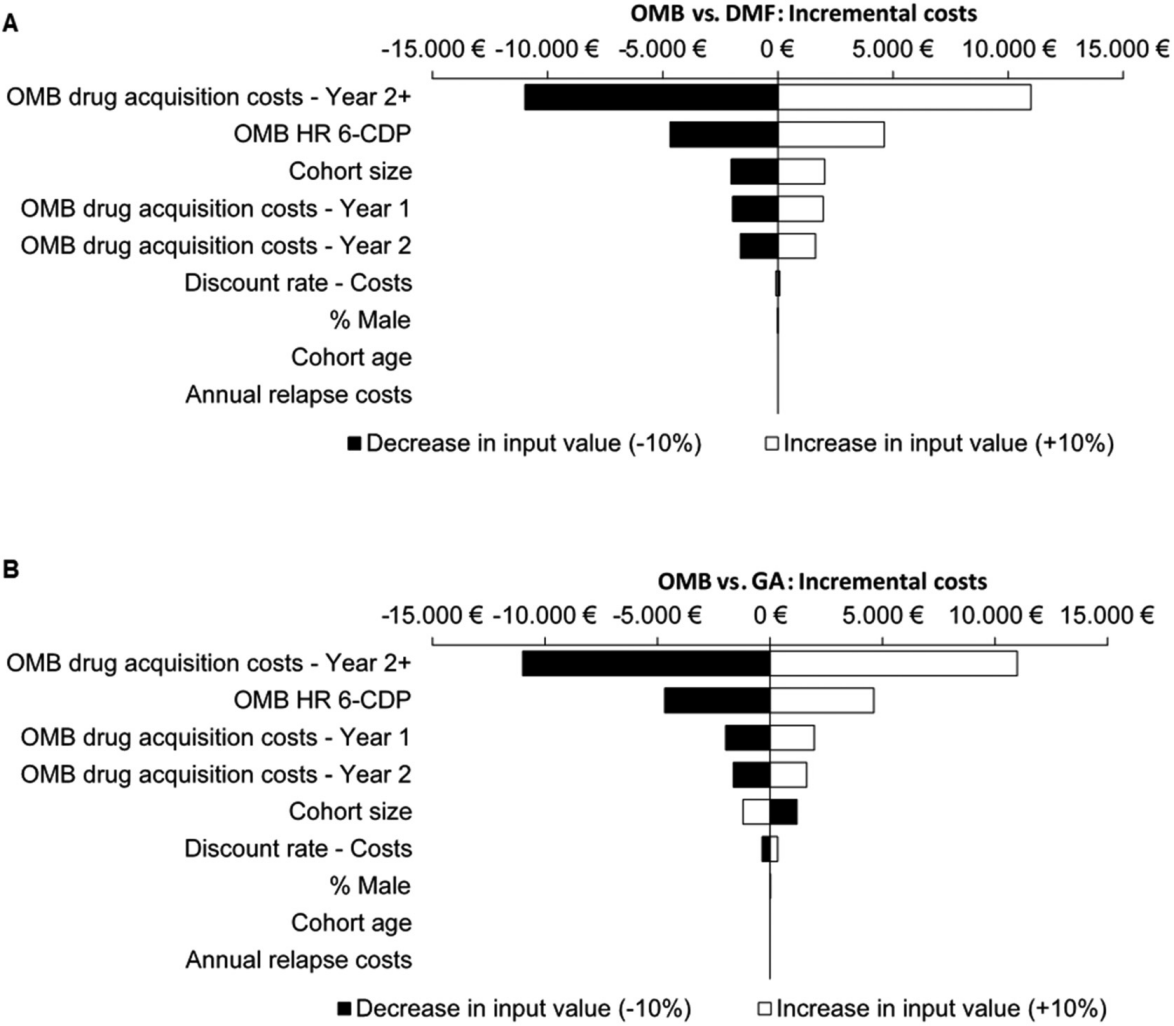
Sensitivity analysis base-scenario: 10 years OMB vs. 10 years DMF (A); 10 years OMB vs. 10 years GA (B); 6-CDP, 6-month confirmed disability progression; DMF, Dimethyl fumarate; EDSS, Expanded Disability Status Scale; GA, Glatiramer acetate; HR, Hazards ratio; OMB, Ofatumumab.

Sensitivity analyses showed that the incremental costs were primarily influenced by DMT costs from the second year onwards. In addition, the results are particularly sensitive to the hazard ratio of 6-CDP. Variations in annual relapse costs as well as age and gender distribution did not impact the results (Figure 3; Figure 4S and 5S).

## Discussion

Over 10 years, immediate OMB treatment or early switch after standard therapy with DMF or GA was estimated to delay EDSS progression, reduce relapse rates, and result in fewer DALYs, less days with informal care as well as higher productivity compared to prolonged standard DMTs. Higher DMT costs associated with immediate or early OMB treatment were compensated by lower additional direct and indirect costs compared to prolonged standard DMTs. The model produced stable and plausible estimates and was insensitive to variables known to have no impact on total costs, e.g., gender.^25^

Our results are in line with clinical studies and real-world evidence reporting a reduced risk of disability progression with highly effective compared to standard DMTs.^7,10,26–28^ The timing of highly effective treatment initiation impacts outcomes. Early initiation delays EDSS progression compared to escalation strategies.^8^ According to data from the international MSBase and Swedish MS registry, 10 years after treatment onset, patients with an early switch to highly effective treatment had an almost stable mean EDSS score, while late switchers showed EDSS worsening of +1.4 and a three-fold increase in the proportion of immobile patients (EDSS ≥ 7).^9^ Likewise, the present model showed almost stable EDSS scores and lower proportions of immobile patients in the immediate and early OMB groups after 10 years compared to late and no switch scenarios. Hence, the results of He et al.^9^ underline the robustness of the model outcomes and indicate that results on immediate or early OMB treatment are similar to real-world settings. Furthermore, the results of He et al.^9^ highlight that an early switch is a relevant scenario in clinical practice. Although supported by the Multiple Sclerosis Therapy Consensus Group,^29^ an immediate use of highly effective DMTs is still linked to a highly active disease course and poor prognosis in current treatment recommendations.^5,6^ For example, the German S2k guideline, which had been revised in May 2021, recommends a switch strategy to highly effective DMTs including anti-CD20 antibodies after initial standard treatment.^6^ Consequently, with early switches being superior to late switches in the present simulation and as long as immediate high efficacious therapy has not yet been established, early switch scenarios will be relevant in clinical practice.

In our model, the lowest number of DALYs were estimated in the immediate OMB cohort. Differences in DALYs between the scenarios were driven by YLDs. No differences were found with regard to YLL, which might be explained by a low mortality ratio of 1.7 used in the model for all EDSS states.^23^ The lower number of DALYs in the immediate OMB and the early switch cohorts were associated with lower EDSS states. This is consistent with a DALY-based estimate of disease burden in Swiss MS patients, in which patients with an EDSS score <4 (68.4% of the total MS population) contributed only 39.8% to the total MS-specific YLD.^30^

The estimated number of relapses and the relapse-related costs in the model were lower in the immediate OMB and early switch cohorts than in the late and no switch cohorts. As two thirds of relapse costs can be attributed to indirect costs,^31^ relapse-related absenteeism, early retirement and invalidity also need consideration. The model revealed the potential of immediate or early OMB treatment to reduce societal burden through better productivity. Furthermore, the model clearly shows that higher DMT costs can be offset when accounting for direct and indirect costs.

The present simulation showed considerable effects on clinical and productivity outcomes of immediate or early treatment with OMB within 10 years. Long-term economic analyses suggest that the beneficial effects will increase over a time horizon of 20 years or more.^32–34^ With increasing disease duration, informal care, inpatient care, and long-term absenteeism become more important due to accumulation of disability. Consequently, the share of drug costs in the total cost assumption will be reduced. It can be expected that over several decades, an early OMB initiation strategy might become both clinically and economically superior compared to prolonged standard DMT.

The Markov model used was EDSS-based, which bears some limitations. The EDSS focusses on functional mobility and is insensitive to impairments such as cognition.^35^ Nevertheless, no composite measures were used, as an EDSS-based definition delivers reproducible results while being less complex. Inherent limitations of Markov models are constant transition probabilities for each cycle and constant efficacy parameters regardless of individual disease course. As this applies to all cohorts, a bias is not expected. The model might underestimate small changes as it used integer EDSS values for modeling tractability and consistency with published literature. As the CCA uses data from various sources, differences between underlying studies bear further limitations. For example, input data on transition probabilities are based on a longitudinal dataset from 1980–1995 and may be outdated. Efficacy data had to be derived from a NMA with adjusted outcomes and short study periods due to a lack of direct comparative data. However, the study populations in the NMA were sufficiently similar to allow for indirect comparison and sufficiently represent the cohort of interest to allow for application in the model. Due to remaining uncertainties of the indirect approach, it remains unclear, whether the observed small differences between the immediate OMB cohort and the early switch scenario will translate into relevant differences in clinical practice. Nevertheless, the considerable differences between estimates for immediate or early OMB and late or no switch cohorts allow the assumption of relevant differences in practice. Probabilities for DMT discontinuation and a possible decrease in effectiveness were not imputed due to a lack of adequate long-term data. This might explain discrepancies between the outcomes reported by He et al.^9^ and our model estimates. Furthermore, the magnitude of difference in proportion of patients in different EDSS states is influenced by the baseline EDSS distribution of patients. The model included RMS patients only while the cost inputs from Flachenecker et al.^3^ included data of primary progressive MS (PPMS) patients. This bias is assumed to be negligible because differences between RMS and PPMS are mainly based on DMT costs, which are calculated separately. In addition, cost input data were based on patients mainly on mildly/moderately efficacious DMTs and thus an overestimation of costs for patients on highly efficacious drugs is possible.^3^

In conclusion, this simulation indicates that immediate OMB treatment or an early switch to OMB results in an overall better health state and higher productivity over 10 years than a late (after 5 years) or no switch from standard DMTs. The clinical benefit of immediate and early OMB treatment together with the approximate cost neutrality from a societal perspective was demonstrated. While medical and patient-related reasons are the main drivers for treatment decisions, from the payer perspective cost neutrality in the long-term becomes relevant. It can be assumed that immediate or early treatment with highly effective DMTs improves the disease course without causing additional costs and should be considered in RMS patients.

## Supplemental Material

sj-pdf-1-mso-10.1177_20552173221085741 - Supplemental material for Comparing the long-term clinical and economic impact of ofatumumab versus dimethyl fumarate and glatiramer acetate in patients with relapsing multiple sclerosis: A cost-consequence analysis from a societal perspective in Germany Click here for additional data file. Supplemental material, sj-pdf-1-mso-10.1177_20552173221085741 for Comparing the long-term clinical and economic impact of ofatumumab versus dimethyl fumarate and glatiramer acetate in patients with relapsing multiple sclerosis: A cost-consequence analysis from a societal perspective in Germany by Dominik Koeditz, Juergen Frensch, Martin Bierbaum, Nils-Henning Ness, Benjamin Ettle, Umakanth Vudumula, Kapil Gudala, Nicholas Adlard, Santosh Tiwari and Tjalf Ziemssen in Multiple Sclerosis Journal – Experimental, Translational and Clinical

## References

[1] Kip M Zimmermann A Krankheitsbild Multiple Sklerose. In: Kip M Schoenfelder T Bless H (eds) Weißbuch Multiple Sklerose in Deutschland. 1st ed. Springer Berlin Heidelberg, 2016.

[2] Kobelt G Thompson A Berg J , et al. New insights into the burden and costs of multiple sclerosis in Europe. Mult Scler 2017; 23: 1123–1136. 28273775 10.1177/1352458517694432 PMC5476197

[3] Flachenecker P Kobelt G, Berg J, et al. New insights into the burden and costs of multiple sclerosis in Europe: results for Germany. Mult Scler 2017; 23(2_suppl): 78–90. 28643593 10.1177/1352458517708141

[4] Kip M Wiendl H . Therapie der Multiplen Sklerose. In: Kip M Schoenfelder T Bless HH (eds) Weißbuch Multiple Sklerose in Deutschland. 1st ed. Springer Berlin Heidelberg, 2016.

[5] Montalban X Gold R Thompson AJ , et al. ECTRIMS/EAN guideline on the pharmacological treatment of people with multiple sclerosis. Mult Scler 2018; 24: 96–120. 29353550 10.1177/1352458517751049

[6] Hemmer B Bayas A Berthele A , et al. Diagnose und Therapie der Multiplen Sklerose, Neuromyelitis-optica-Spektrum-Erkrankungen und MOG-IgG-assoziierten Erkrankungen, S2k-Leitlinie. Leitlinien für Diagnostik und Therapie in der Neurologie: Deutsche Gesellschaft für Neurologie. 2021.

[7] Brown JWL Coles A Horakova D , et al. Association of initial disease-modifying therapy with later conversion to secondary progressive multiple sclerosis. JAMA 2019; 321: 175–187. 30644981 10.1001/jama.2018.20588 PMC6439772

[8] Harding K Williams O Willis M , et al. Clinical outcomes of escalation vs early intensive disease-modifying therapy in patients with multiple sclerosis. JAMA Neurol 2019; 76: 536–541. 30776055 10.1001/jamaneurol.2018.4905 PMC6515582

[9] He A Merkel B Brown JWL , et al. Timing of high-efficacy therapy for multiple sclerosis: a retrospective observational cohort study. Lancet Neurol 2020; 19: 307–316. 32199096 10.1016/S1474-4422(20)30067-3

[10] Buron MD Chalmer TA Sellebjerg F , et al. Initial high-efficacy disease-modifying therapy in multiple sclerosis: a nationwide cohort study. Neurology 2020; 95: e1041–e51. 32636328 10.1212/WNL.0000000000010135

[11] Ziemssen T Derfuss T de Stefano N , et al. Optimizing treatment success in multiple sclerosis. J Neurol 2016; 263: 1053–1065. 26705122 10.1007/s00415-015-7986-y PMC4893374

[12] Bar-Or A Grove RA Austin DJ , et al. Subcutaneous ofatumumab in patients with relapsing-remitting multiple sclerosis: the MIRROR study. Neurology 2018; 90: e1805–e14. 29695594 10.1212/WNL.0000000000005516 PMC5957306

[13] Genovese MC Kaine JL Lowenstein MB , et al. Ocrelizumab, a humanized anti-CD20 monoclonal antibody, in the treatment of patients with rheumatoid arthritis: a phase I/II randomized, blinded, placebo-controlled, dose-ranging study. Arthritis Rheum 2008; 58: 2652–2661. 18759293 10.1002/art.23732

[14] Hauser SL Bar-Or A Cohen JA , et al. Ofatumumab versus teriflunomide in multiple sclerosis. N Engl J Med 2020; 383: 546–557. 32757523 10.1056/NEJMoa1917246

[15] Hauser SL Bar-Or A Comi G , et al. Ocrelizumab versus interferon beta-1a in relapsing multiple sclerosis. N Engl J Med 2017; 376: 221–234. 28002679 10.1056/NEJMoa1601277

[16] Gartner J Hauser S Bar-Or A , et al. MSVirtual 2020—poster abstracts. P0192. Benefit-risk of ofatumumab in treatment-naïve early relapsing multiple sclerosis patients. Mult Scler 2020; 26(3_suppl): 118–659.30541380

[17] FDA. Drugs@FDA: FDA-Approved Drugs. Biologic License Application (BLA): 125326 2020 [updated 20.08.2020]. [last accessed 14.01.2022] https://www.accessdata.fda.gov/scripts/cder/daf/index.cfm?event=overview.process&ApplNo=125326.

[18] EMA. Kesimpta. Ofatumumab 2021 [updated 12.05.2021, https://www.ema.europa.eu/en/medicines/human/EPAR/kesimpta.

[19] Ness NH Haase R Kern R , et al. The multiple sclerosis health resource utilization survey (MS-HRS): development and validation study. J Med Internet Res 2020; 22: e17921. 32181745 10.2196/17921 PMC7109610

[20] Mauskopf JA Paul JE Grant DM , et al. The role of cost-consequence analysis in healthcare decision-making. Pharmacoeconomics 1998; 13: 277–288. 10178653 10.2165/00019053-199813030-00002

[21] Samjoo IA Worthington E Drudge C , et al. Comparison of ofatumumab and other disease-modifying therapies for relapsing multiple sclerosis: a network meta-analysis. J Comp Eff Res 2020; 9: 1255–1274. 33090003 10.2217/cer-2020-0122

[22] Statistisches Bundesamt. Sterbetafeln: Ergebnisse aus der laufenden Berechnung von Periodensterbetafeln für Deutschland und die Bundesländer: 2017/2019.2020, https://www.destatis.de/DE/Themen/Gesellschaft-Umwelt/Bevoelkerung/Sterbefaelle-Lebenserwartung/_inhalt.html (accessed 14 October 2020).

[23] Jick SS Li L Falcone GJ , et al. Mortality of patients with multiple sclerosis: a cohort study in UK primary care. J Neurol 2014; 261: 1508–1517. 24838537 10.1007/s00415-014-7370-3 PMC4119255

[24] Ness NH Schriefer D Haase R , et al. Real-world evidence on the societal economic relapse costs in patients with multiple sclerosis. Pharmacoeconomics 2020; 38: 883–892. 32363542 10.1007/s40273-020-00917-3

[25] Schriefer D Ness NH Haase R , et al. Gender disparities in health resource utilization in patients with relapsing-remitting multiple sclerosis: a prospective longitudinal real-world study with more than 2000 patients. Ther Adv Neurol Disord 2020; 13: 1756286420960274. 33178335 10.1177/1756286420960274 PMC7592171

[26] Hutchinson M Kappos L Calabresi PA , et al. The efficacy of natalizumab in patients with relapsing multiple sclerosis: subgroup analyses of AFFIRM and SENTINEL. J Neurol 2009; 256: 405–415. 19308305 10.1007/s00415-009-0093-1

[27] Cohen JA Barkhof F Comi G , et al. Oral fingolimod or intramuscular interferon for relapsing multiple sclerosis. N Engl J Med 2010; 362: 402–415. 20089954 10.1056/NEJMoa0907839

[28] Coles AJ Compston DA Selmaj KW , et al. Alemtuzumab vs. interferon beta-1a in early multiple sclerosis. N Engl J Med 2008; 359: 1786–1801. 18946064 10.1056/NEJMoa0802670

[29] Wiendl H Gold R Berger T , et al. Multiple sclerosis therapy consensus group (MSTCG): position statement on disease-modifying therapies for multiple sclerosis (white paper). Ther Adv Neurol Disord 2021; 14: 17562864211039648. 34422112 10.1177/17562864211039648 PMC8377320

[30] Kaufmann M Puhan MA Salmen A , et al. 60/30: 60% of the morbidity-associated multiple sclerosis disease burden comes from the 30% of persons with higher impairments. Front Neurol 2020; 11: 156. 32210908 10.3389/fneur.2020.00156 PMC7068809

[31] Ness NH Schriefer D Haase R , et al. Differentiating societal costs of disability worsening in multiple sclerosis. J Neurol 2020; 267: 1035–1042. 31848738 10.1007/s00415-019-09676-4

[32] Frasco MA Shih T Incerti D , et al. Incremental net monetary benefit of ocrelizumab relative to subcutaneous interferon β-1a. J Med Econ 2017; 20: 1074–1082. 28726530 10.1080/13696998.2017.1357564

[33] Furneri G Santoni L Ricella C , et al. Cost-effectiveness analysis of escalating to natalizumab or switching among immunomodulators in relapsing-remitting multiple sclerosis in Italy. BMC Health Serv Res 2019; 19: 436. 31253138 10.1186/s12913-019-4264-1 PMC6599237

[34] Yang H Duchesneau E Foster R , et al. Cost-effectiveness analysis of ocrelizumab versus subcutaneous interferon beta-1a for the treatment of relapsing multiple sclerosis. J Med Econ 2017; 20: 1056–1065. 28703659 10.1080/13696998.2017.1355310

[35] Inojosa H Schriefer D Ziemssen T . Clinical outcome measures in multiple sclerosis: a review. Autoimmun Rev 2020; 19: 102512. 32173519 10.1016/j.autrev.2020.102512

