# Supplementary material for: Comparing the long-term clinical and economic impact of ofatumumab versus dimethyl fumarate and glatiramer acetate in patients with relapsing multiple sclerosis: A cost-consequence analysis from a societal perspective in Germany
Source: Mult Scler J Exp Transl Clin. 2022 Mar 29;8(1):20552173221085741. doi: 10.1177/20552173221085741 (PMC8969034; doi:10.1177/20552173221085741)
Supplement: sj-pdf-1-mso-10.1177_20552173221085741 - Supplemental material for Comparing the long-term clinical and economic impact of ofatumumab versus dimethyl fumarate and glatiramer acetate in patients with relapsing multiple sclerosis: A cost-consequence analysis from a societal perspective in Germany [file sj-pdf-1-mso-10.1177_20552173221085741.pdf]

Supplement

Model structure

The EDSS-based, discrete-time Markov model comprised a natural history reference model (off-treatment) including data on disability progression of RMS patients treated with best supportive care (BSC) and a treatment-adjusted model combining the natural history reference model with data on the comparative outcomes of different DMTs (on-treatment). The Markov model included 11 health states of the EDSS, ranging from 0 (no disability) to 9 representing disease progression and a single state (EDSS 10) for death from all causes. The health states were defined as 1-point increments of integer EDSS values (intermediate values were rounded down), the cycle length was one year. Outcomes were evaluated over a time horizon of 10 years. Patients with RMS entered the model in one of the 10 EDSS states. According to the transition probabilities resulting from the results on the effectiveness of the respective treatments, the cohort was exposed to the following risks in each cycle: (1) EDSS progression (transition to a higher EDSS state), EDSS improvement (transition to a lower EDSS state), stable EDSS (remaining in the current EDSS state) and DMT-discontinuation criteria based on EDSS score (EDSS >6.0), relapse or death. No distinction was made between RRMS and SPMS as SPMS is usually diagnosed retrospectively and the timepoint of transition is difficult to determine. It was assumed that patients progressing to SPMS remaining on treatment (exception: EDSS score ≥7), have the same transition probabilities as RRMS patients. In general, interventions with DMTs were assumed to be associated with a reduction in the number of relapses and a slowing of disability progression. A simplified structure of the model is represented in Figure S1. For treatment discontinuers, the benefits achieved with the respective DMTs are accumulated until treatment discontinuation. From that point on, progression and relapse rates according to BSC were applied.

Input variables

Patient characteristics of the target population: The treatment-naïve subpopulation of the combined ASCLEPIOS I and II phase 3 trials were used for data input at baseline (1). This previously untreated population included 615 patients with RRMS or SPMS (Table S1).

Data for the natural history model: The transition probabilities between EDSS states of the natural, off-treatment history model were based on the dataset reported by Palace and colleagues (2), which was derived from 898 untreated RMS patients in the British Columbia Multiple Sclerosis (BCMS) database during the years 1980-1995. The calculation of annual relapse rates (ARR) by EDSS states during the untreated course of the disease were based on a study of British MS patients (3) and a prospective long-term study by Patzold & Pocklington (4) (Table S2).

Data for the treatment-adjusted model: The transition probabilities for the on-treatment model were modified by the reduction of disability progression achieved with the respective DMT treatment at the approved standard dose (OMB 20 mg every month, dimethyl fumarate 240 mg twice per day, or glatiramer acetate 20 mg every day). The central parameter for modelling disease progression was the 6-month CDP, expressed as a hazard ratio. Therapy-specific inputs including 6-month CDPs and ARR were derived from a network meta-analysis (NMA) (5). An NMA estimates the comparative efficacy and/or safety of a number of interventions. Typically, a systematic review is used to assemble all trial evidence of interest into an evidence network that will inform the NMA. At this stage, the comparability of populations, duration, outcome definitions and the feasibility of the statistical analysis for the NMA is assessed. The reported differences in the outcome measure between interventions and corresponding measure of uncertainty in each trial are combined using Monte-Carlo Markov chain methods. In this way the benefit of randomization in each source study is preserved when undertaking the network meta-analysis. For the present purpose, a NMA on the efficacy of existing RRMS therapies and OMB was performed including the results of the ASCLEPIOS trials on OMB. The resulting NMA has been published by Samjoo et al. (5). The included studies recruited patients with RMS aged between 18 and 65 years. Mean age ranged from 30 to 40 years, mean time since first symptoms was 2 to 10 years, and mean baseline EDSS ranged from 2 to 3 among the NMA studies (5). The study populations in the NMA were sufficiently similar to allow for indirect comparison within the NMA. Furthermore, the NMA study populations sufficiently cover the ASCLEPIOS treatment-naÏve subpopulations included as baseline model input data and sufficiently represent the cohort of interest over the 10 year time horizon to allow for application in the model. CDP and ARR results used for the present model are presented in Table S2.

Mortality and disability weights: Mortality rates of the general population were taken from the mortality tables of the German Federal Statistical Office (6), stratified for gender and age and adjusted using the constant mortality hazard ratios reported in the MS population (7). MS-specific disability weights were determined by Cho and colleagues, based on a survey of MS experts, using trade-off procedures per EDSS score (Table S3). The severity of MS and associated health impairments are expressed on a scale from 0 (perfect health) to 1 (death) (8).

Productivity loss: Data on MS-related productivity loss were derived from a multinational study on the burden and costs of MS (9) and German registry data (10). Productivity loss inputs were the proportion of all employees, full employees, patients receiving invalidity pension and the number of days of informal care per year and patient (Table S4). Retirement age and working days per year were assumed as 67 years and 260 days, respectively.

Costs: Direct costs and indirect costs were considered regardless of the responsible payer. Direct costs included healthcare costs (DMT costs, inpatient care, day case admissions, consultations, tests, and medications other than DMTs), as well as services and informal costs (community and social services, investments and equipment, and informal care). Indirect costs comprised costs for short- and long-term absences from work, invalidity and early retirement. Annual disease-related costs per patient were obtained from the multinational MS study and stratified according to EDSS state (11). On the basis of consumer price indices data were inflated to the year 2020. Annual drug acquisition costs were based on the German pharmacy prices schedule for dimethyl fumarate and glatiramer acetate. The annual treatment costs for OMB were assumed to be in the range of first-line therapies (Table S5). Annual relapse costs were based on the costs reported by Ness and colleagues (12). For this purpose the quarterly reported relapse costs of Ness and colleagues were upscaled to annual relapse costs. The annual relapse costs per patient were estimated at 2,662 €, taking into account the consumer price indices (Table S6). The costs for monitoring and adverse events over time were marginal with relatively limited impact on the total costs and were therefore not considered in this analysis.

To estimate the average number of patients in a given Markov state, the life table method was used, which averages the number of patients in the beginning of the cycle and after the transitions to new

EDSS scores (13, 14). Costs and effects were discounted annually at 3.0% in accordance with the recommendation of the Institute for Quality and Efficiency in Health Care (IQWiG) (15).

Model assumptions

Treatment effects: Patients were assumed as fully adherent prior to discontinuation of therapy. The treatment effects and transition probabilities were considered to remain constant over time, without decrease in treatment effectiveness over time. Treatment with DMTs was associated with the assumption that patients benefit in terms of delaying progression and reducing annual relapse rates.

Discontinuation of DMTs: Patients were assumed to discontinue treatment when they reached an EDSS score of ≥7 or transitioned to SPMS instead of applying treatment-specific discontinuation rates for the following reasons: 1) Discontinuation of therapy at EDSS score of ≥7 is recommended by some guidelines. 2) The long-term discontinuation rates of OMB treatment were not known and can only be derived from other therapies to a limited extent. 3) Discontinuation rates observed in clinical trials were representative during the first year, but are subsequently considered far too high, especially with OMB as an induction therapy.

Calculation of Model outputs: The assignment to an EDSS health state was the central determinant for the calculation of clinical and economic outcomes.

Tables to supplement

Table S1: Demographic & disease characteristics of treatment-naïve patients	
Mean age - years	36.3±9.23
Male sex - %	33.0%
Type of disease	
• RRMS	98.7%
• SPMS	1.3%
Duration of MS since diagnosis - years	0.56±0.576
Duration of MS since first symptom - years	3.33±4.115
Number of relapses in the last 12 months prior to screening	1.4±0.71
Time since onset of the most recent relapse - months	6.12±11.67
T2 lesion volume, cm^3	9.2±10.72
Patients free of GD+ T1 lesions - %	55.9%
Number of GD+ T1 lesions	1.6±3.66
**Distribution over EDSS states, %**	
EDSS 0	4.9%
EDSS 1	31.9%
EDSS 2	31.9%
EDSS 3	18.5%
EDSS 4	9.4%
EDSS 5	3.1%
EDSS 6	0.3%
EDSS 7	0.0%
EDSS 8	0.0%
EDSS 9	0.0%
Mean EDSS-Score	2.3±1.2

Source: treatment-naïve patients of ASCLEPIOS I and II trials (1) N=615; EDSS, Expanded Disability Status Scale; MS, Multiple Sclerosis; RRMS, Relapsing Remitting MS; SPMS, Secondary Progressive MS; GD, Gadolinium.

**Table S2: Annualized Relapse Rate (=ARR) & Time to Confirmed Disability Progression (6-CDP)**											
Natural History reference model											
EDSS	0	1	2	3	4	5	6	7	8	9	Source
ARR*	0.71	0.73	0.68	0.72	0.71	0.59	0.49	0.47	0.51	0.52	(3, 4)
CDP*	Transition probabilities based on British Columbia Natural History dataset										(2)
Treatment-adjusted model											
Therapy				ARR				CDP-6			
OMB				0.30				0.43			(5)
DMF				0.50				0.68			
GA				0.65				0.78			

*ARRs and CDPs by EDSS state were calculated by combining data of the of British MS patients (3) and a prospective long-term study by Patzold & Pocklington (4); ARR, Annualized Relapse Rate; CDP, Confirmed Disability Progression; EDSS, Expanded Disability Status Scale; DMF, Dimethyl fumarate; GA, Glatiramer acetate; OMB, Ofatumumab.

Table S3: Productivity loss

	Employed or self-employed, %[a]	Working full time, %[b]	Invalidity pension, %[a]	Source
EDSS 0	82.0	37.9	18.5	(9, 11)
EDSS 1	77.0	37.9	18.5	
EDSS 2	68.0	37.9	18.5	
EDSS 3	54.0	37.9	18.5	
EDSS 4	49.0	33.1	48.2	
EDSS 5	39.0	33.1	48.2	
EDSS 6	28.5	33.1	48.2	
EDSS 7	16.0.	32.1	64.2	
EDSS 8	15.0	32.1	64.2	
EDSS 9	8.0	32.1	64.2	

[a] Proportion of patients below retirement age.
[b] Proportion of patients working.
Data on file for number of days of informal care per year and patient. Data were obtained from Flachenecker et al. to be used as model inputs only.

Table S4: Disability weights

	Weight	Source
EDSS 0	0.000	(8)
EDSS 1	0.011	
EDSS 2	0.021	
EDSS 3	0.110	
EDSS 4	0.199	
EDSS 5	0.256	
EDSS 6	0.313	
EDSS 7	0.617	
EDSS 8	0.772	
EDSS 9	0.926	

Table S5: Drug acquisition costs

Therapy	Pack	Unit/ pack	Unit cost	Units consumed per year 1 / 2+	Total cost year 1*	Total cost year 2+*	Source
OMB	3 x 0,4 ml	3	1,411,17€	14 / 12	19.756€	16.934€	Lauer-Taxe® Extavia (02/21)
DMF	12 x 240 mg oral	168	15,43€	730.00 / 730.00	11.267€	11.267€	Lauer-Taxe® (02/21)
GA	40mg injection solution	36	87,80€	156.43 / 156.43	13.735€	13.735€	Lauer-Taxe® (02/21)

*Annual therapy costs based on net pharmacy retail price; most economical pack size. DMF, Dimethyl fumarate; GA, Glatiramer acetate; OMB, Ofatumumab.

Table S6: Disease related costs by EDSS Score & annual relapse costs (*Mean annual cost per patient in 2020 in Euro)*

	EDSS										Source
	0	1	2	3	4	5	6	7	8	9	
**Direct costs**											(11)
Healthcare costs[a] *(Inpatient care, Day admission, Consultations, Tests, Medications)*[b]	1,439	1,985	3,820	5,012	6,068	7,131	8,905	11,051	12,201	25,972	
Services & informal care[a] *(Community services, Investments, Informal care)*[b]	219	482	1,389	2,365	3,820	6,634	10,442	21,280	35,341	45,991	
**Indirect costs**[a] *(Short term absence, Long-term absence invalidity, early retirement)*[b]	3,533	4,823	10,230	14,786	17,330	19,726	21,424	23,823	22,966	33,777	
**Relapse costs**	2.662										(12)

a: Projection of costs for 2020 based on 2015 data from Flachenecker et al. (11).
b: Data on file for disaggregated costs per subordinate categories. Data on subordinate costs were obtained from Flachenecker et al. to be used as model inputs only.
EDSS, Expanded Disability Status Scale.

Figures to supplement

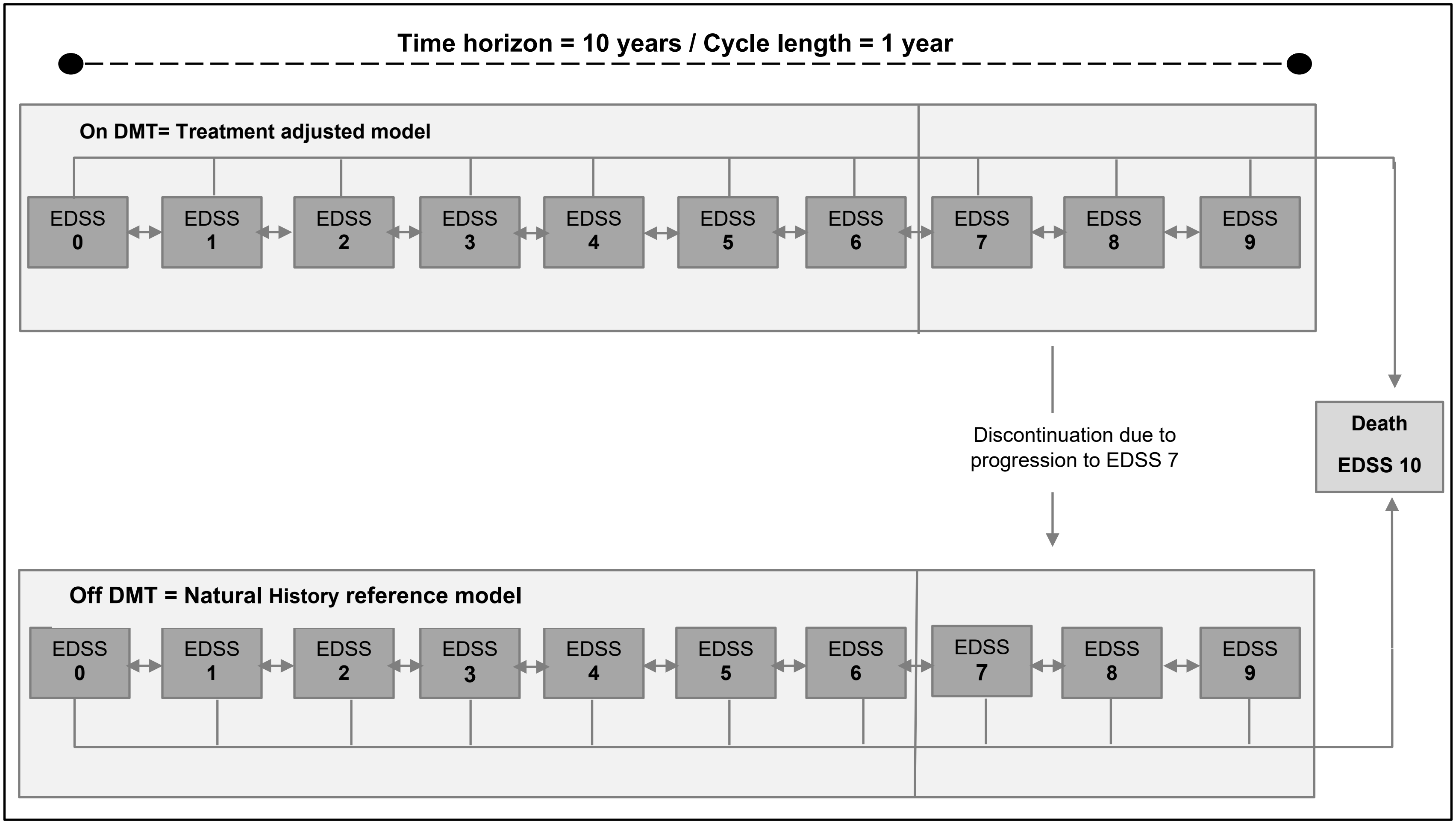

DMT, Disease-modifying therapy; EDSS, Expanded Disability Status Scale.

Figure S1: Health state structure of the 11-state Markov Model

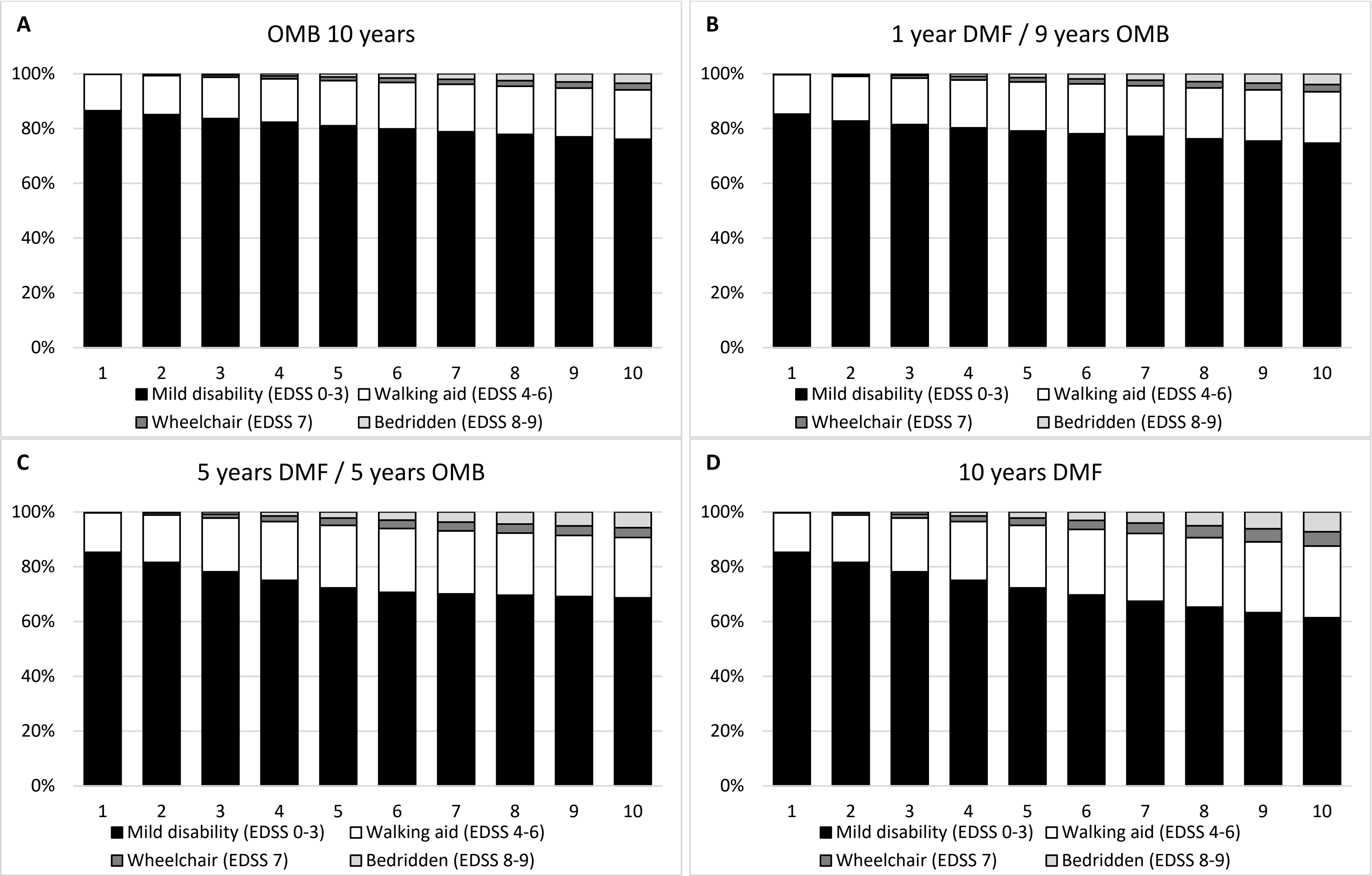

Figure S2: EDSS distribution over 10 years: 10 years OMB (A); 1 year DMF followed by 9 years OMB (B); 5 years DMF followed by 5 years OMB (C); 10 years DMF (D).

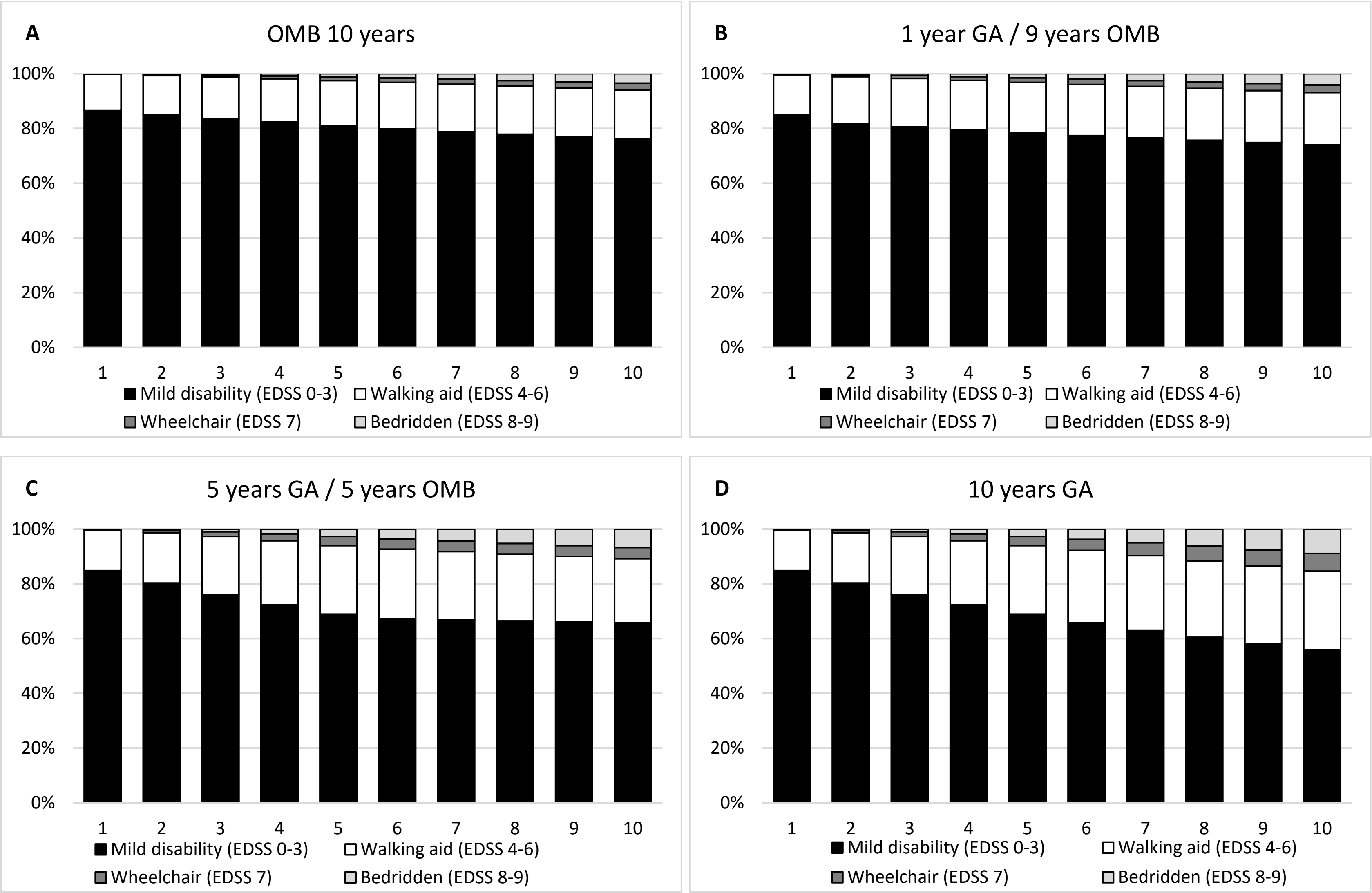

Figure S3: EDSS distribution over 10 years: 10 years OMB (A); 1 year GA followed by 9 years OMB (B); 5 years GA followed by 5 years OMB (C); 10 years GA (D).

A

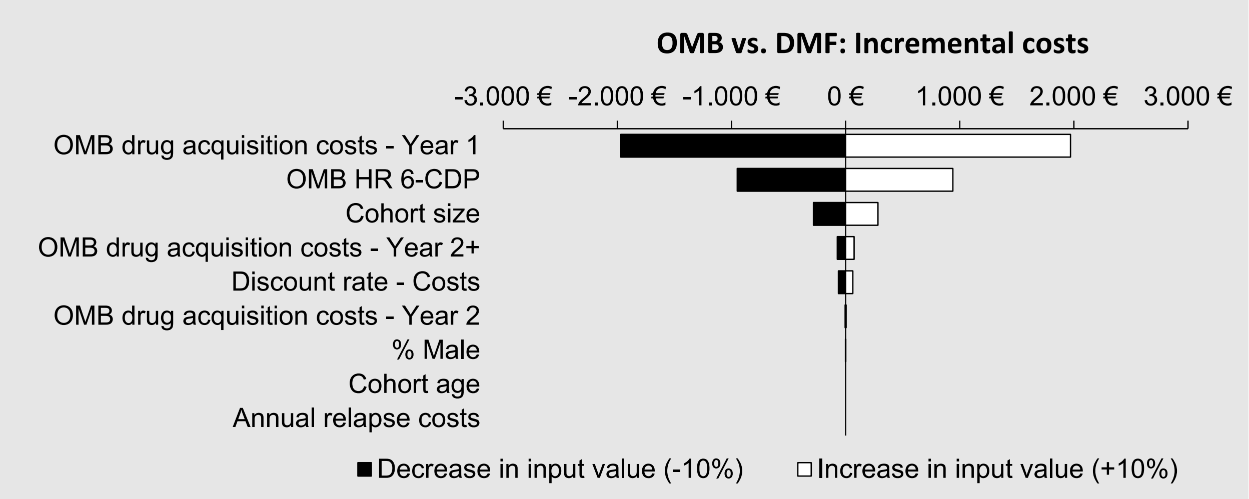

B

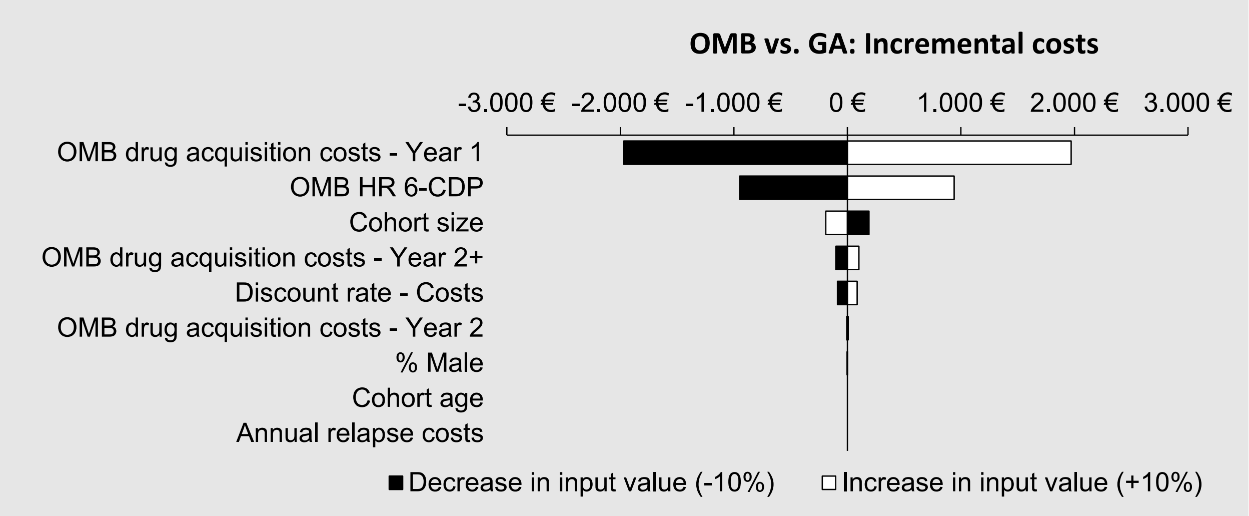

6-CDP, 6-month Confirmed Disability Progression; EDSS, Expanded Disability Status Scale; DMF, Dimethyl fumarate; GA, Glatiramer acetate; HR, Hazards ratio; OMB, Ofatumumab.

Figure S4: Sensitivity analysis for scenario A: 10 years OMB vs. 1 year DMF followed by 9 years OMB (A); 10 years OMB vs. 1 year GA followed by 9 years OMB (B).

A

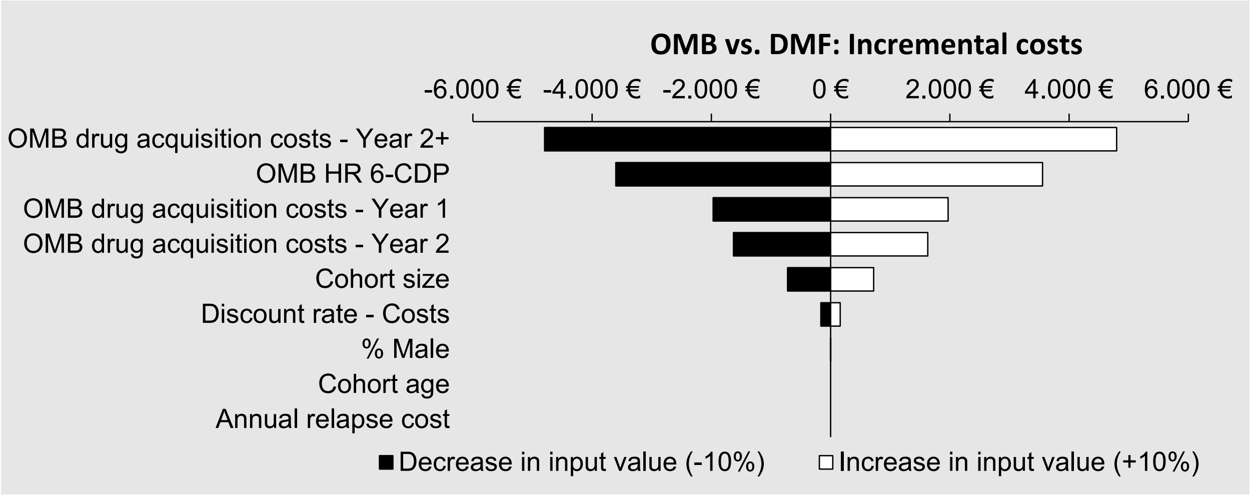

B

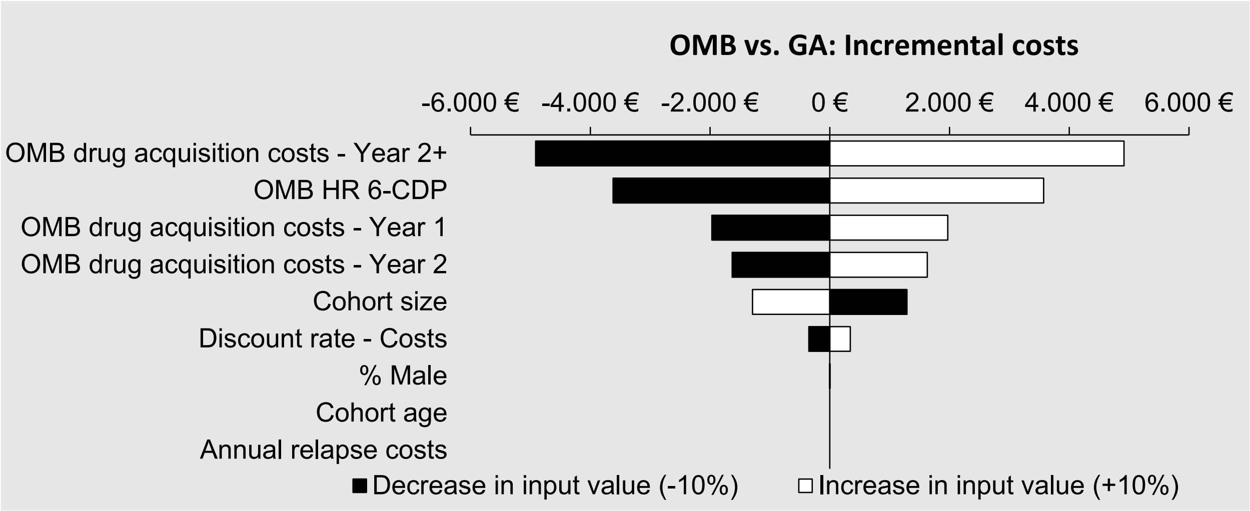

6-CDP, 6-month Confirmed Disability Progression; DMF, Dimethyl fumarate; GA, Glatiramer acetate; HR, Hazards ratio; OMB, Ofatumumab.

Figure S5: Sensitivity analysis for scenario B: 10 years OMB vs. 5 years DMF followed by 5 years OMB vs. (A); 10 years OMB vs. 5 years GA followed by 5 years OMB (B).

References to supplement

1. Gartner J, Hauser S, Bar-Or A, Montalban X, Cohen J, Cross AH, et al. MSVirtual 2020 - Poster Abstracts. P0192. Benefit-risk of ofatumumab in treatment-naïve early relapsing multiple sclerosis patients. Mult Scler. 2020;26(3_suppl):118-659.
2. Palace J, Bregenzer T, Tremlett H, Oger J, Zhu F, Boggild M, et al. UK multiple sclerosis risk-sharing scheme: a new natural history dataset and an improved Markov model. BMJ Open. 2014;4(1):e004073.
3. Orme M, Kerrigan J, Tyas D, Russell N, Nixon R. The effect of disease, functional status, and relapses on the utility of people with multiple sclerosis in the UK. Value Health. 2007;10(1):54-60.
4. Patzold U, Pocklington PR. Course of multiple sclerosis. First results of a prospective study carried out of 102 MS patients from 1976-1980. Acta Neurol Scand. 1982;65(4):248-66.
5. Samjoo IA, Worthington E, Drudge C, Zhao M, Cameron C, Haring DA, et al. Comparison of ofatumumab and other disease-modifying therapies for relapsing multiple sclerosis: a network meta-analysis. J Comp Eff Res. 2020;9(18):1255-74.
6. Statistisches Bundesamt. Sterbetafeln: Ergebnisse aus der laufenden Berechnung von Periodensterbetafeln für Deutschland und die Bundesländer: 2017/2019.2020 Accessed October 14, 2020. Available from: https://www.destatis.de/DE/Themen/Gesellschaft-Umwelt/Bevoelkerung/Sterbefaelle-Lebenserwartung/_inhalt.html.
7. Jick SS, Li L, Falcone GJ, Vassilev ZP, Wallander MA. Mortality of patients with multiple sclerosis: a cohort study in UK primary care. J Neurol. 2014;261(8):1508-17.
8. Cho JY, Hong KS, Kim HJ, Kim SH, Min JH, Kim NH, et al. Disability weight for each level of the Expanded Disability Status Scale in multiple sclerosis. Mult Scler. 2014;20(9):1217-23.
9. Kobelt G, Thompson A, Berg J, Gannedahl M, Eriksson J, Group MS, et al. New insights into the burden and costs of multiple sclerosis in Europe. Mult Scler. 2017;23(8):1123-36.
10. Flachenecker P, Eichstadt K, Berger K, Ellenberger D, Friede T, Haas J, et al. [Multiple sclerosis in Germany: updated analysis of the German MS Registry 2014-2018]. Fortschr Neurol Psychiatr. 2020;88(7):436-50.
11. Flachenecker P, Kobelt G, Berg J, Capsa D, Gannedahl M, European Multiple Sclerosis Platform. New insights into the burden and costs of multiple sclerosis in Europe: Results for Germany. Mult Scler. 2017;23(2_suppl):78-90.
12. Ness NH, Schriefer D, Haase R, Ettle B, Ziemssen T. Real-World Evidence on the Societal Economic Relapse Costs in Patients with Multiple Sclerosis. Pharmacoeconomics. 2020;38(8):883-92.
13. Barendregt JJ. The half-cycle correction: banish rather than explain it. Med Decis Making. 2009;29(4):500-2.
14. Barendregt JJ. The life table method of half cycle correction: getting it right. Med Decis Making. 2014;34(3):283-5.
15. Institute for Quality and Efficiency in Health Care (IQWiG). General Methods Version 6.0.2020 Accessed June 15, 2021. Available from: https://www.iqwig.de/ueber-uns/methoden/methodenpapier/.
